# *RHOA* mutation in diffuse-type gastric cancer: a comparative clinicopathology analysis of 87 cases

**DOI:** 10.1007/s10120-015-0493-0

**Published:** 2015-04-01

**Authors:** Tetsuo Ushiku, Shumpei Ishikawa, Miwako Kakiuchi, Atsushi Tanaka, Hiroto Katoh, Hiroyuki Aburatani, Gregory Y. Lauwers, Masashi Fukayama

**Affiliations:** 1 Department of Pathology, Graduate School of Medicine, The University of Tokyo, Tokyo, Japan; 2 Department of Genomic Pathology, Medical Research Institute, Tokyo Medical and Dental University, 1-5-45 Yushima, Bunkyo-ku, Tokyo 113-8510 Japan; 3 Genome Science Division, Research Center for Advanced Science and Technology, The University of Tokyo, Tokyo, Japan; 4 Department of Gastroenterology, Graduate School of Medicine, The University of Tokyo, Tokyo, Japan; 5 Department of Pathology, Massachusetts General Hospital Boston, Boston, MA USA

**Keywords:** *RHOA*, Mutation, Gastric cancer, Diffuse type

## Abstract

**Background:**

Recent studies have discovered recurrent *RHOA* mutations in diffuse-type gastric cancers. These reports show mutant RhoA is an important cancer driver and is a potential therapeutic target. This study aims to investigate the clinicopathological features of diffuse-type gastric cancers with *RHOA* mutation.

**Methods:**

We performed a thorough review of 87 diffuse-type gastric cancers, including 22 *RHOA*-mutated and 65 *RHOA* wild-type gastric cancers.

**Results:**

Most advanced tumors with *RHOA* mutation appeared as Borrmann type 3 lesions (81 %) developing in the middle (50 %) or distal (32 %) third of the stomach. Histologically, although all of the tumors were predominantly or exclusively composed of poorly cohesive carcinoma, limited tubular differentiation was also observed in 73 % of the *RHOA*-mutated tumors. Notably, *RHOA*-mutated tumors more frequently showed a permeative growth pattern at the edge of the mucosal area (59 %) compared with *RHOA* wild-type tumors (29 %, *P* = 0.0202). Additionally, the size ratios of the deeply invasive components to the mucosal components were significantly lower in *RHOA*-mutated tumors [less than 1.45 (median) in 68 % of cases] than in *RHOA* wild-type tumors (less than 1.45 in 42 % of cases, *P* = 0.0482). *RHOA* mutation did not significantly impact survival in this study.

**Conclusions:**

These observations suggest that *RHOA* mutation may be associated with the growth patterns of diffuse-type gastric cancer but have a limited prognostic impact in isolation. Further studies, including analyses of the other alterations involving the RhoA pathways, such as *CLDN18*–*ARHGAP* fusion, as well as functional studies of mutant RhoA, are necessary to clarify the significance of alterations in the RhoA-signaling pathway in diffuse-type gastric cancers.

## Introduction

Gastric cancer remains the third leading cause of cancer death worldwide. Despite improvements in the treatment of gastric cancer, patients with advanced or metastatic disease have a poor prognosis, with 5-year survival rates of approximately 5–15 % for stage IV disease [1, 2]. The combination of chemotherapy and treatment with trastuzumab, which is an antibody against human epidermal growth factor receptor 2 (HER2), is thus far the only proven targeted therapy that is indicated for patients with HER2-positive gastric cancer [3]. Unfortunately, most patients with diffuse-type gastric cancer (according to the Lauren classification [4]) may not receive trastuzumab therapy because the HER2-positivity rates are only 2–7 % in this histological type [5–8]. Therefore, identification of a potential therapeutic target for these patients with aggressive diffuse-type gastric cancer would be particularly valuable.

Several recent studies involving the use of whole-exome or whole-genome sequencing have reported recurrent nonsynonymous mutations of *RHOA* in a subset of gastric cancers [9–11]. *RHOA*, which encodes the small GTPase RhoA, is a master regulator of actin–myosin-dependent cell contractility and cellular motility [12, 13]. Although until recently *RHOA* has never been reported to be mutated in human cancers, its overexpression and association with tumor progression have been reported in various cancers [14–19]. Notably, *RHOA* mutation has been detected almost exclusively in diffuse-type gastric cancers, accounting for 14–25 % of the cases of this type, whereas it is absent in intestinal-type cancers [9–11]. The functional assays in our report using small interfering RNA knockdowns and rescue experiments have demonstrated the growth-promoting effects of mutant RhoA [9]. From these observations, mutant RhoA is likely to function in a gain-of-function manner and play a key role in the carcinogenesis of diffuse-type gastric cancer. Importantly, RhoA could be a potential druggable oncogenic protein because it has various targetable domains, such as binding pockets for GTP and structural regions for protein–protein interactions with effectors, RhoGAPs and RhoGEFs. However, the previous studies have lacked thorough histological descriptions, and the clinicopathological significance of *RHOA* mutation in diffuse-type gastric cancer is largely unknown.

In this study, we performed a retrospective analysis of 87 cases of diffuse-type gastric cancer, including 22 *RHOA*-mutant and 65 *RHOA* wild-type cases, to better clarify the clinicopathological features of *RHOA*-mutated diffuse-type gastric cancer. In light of the previous reports that RhoA is associated with tumor cell motility and invasion in various types of cancers [18, 20, 21], we performed a histological review with a particular focus on the association between the *RHOA* mutation and growth patterns.

## Materials and methods

### Study group

The study was approved by the Institutional Review Board of Tokyo University Hospital. The cohort included 87 patients with diffuse-type gastric cancer with an established mutation status of the *RHOA* gene, which had been determined by whole-exome sequencing (*n* = 30) or targeted deep sequencing (*n* = 57) in our previous study [9]. Twenty-two of the 87 tumors (25 %) were *RHOA* mutated, and the remaining 65 tumors (75 %) were *RHOA* wild type.

### Clinical data

The demographic data, endoscopic findings, and clinical follow-up data were obtained by reviewing the medical records. Tumor staging was performed according to the tumor–node–metastasis classification system [22]. The macroscopic tumor type was classified according to the criteria of the World Health Organization classification for early gastric cancer and the Borrmann classification for advanced gastric cancer [22].

### Histological evaluation

All the assessments were performed on the basis of the examination of the histological sections of the primary tumor by a gastrointestinal pathologist (T.U.) without knowledge of the mutation status and outcome of the patients. The number of hematoxylin and eosin stained sections per tumor ranged from 3 to 36 (mean 12.6; median 11). The histological features were recorded: histological type, stromal features, growth pattern, tumor size, tumor stage, lymphovascular invasion, perineural invasion, and nodal metastasis. The histological type was determined according to the criteria of the World Health Organization classification: tubular, papillary, mucinous, and poorly cohesive carcinomas [22]. Although all the tumors that were included in this study consisted predominantly or exclusively of poorly cohesive carcinoma because this study included only diffuse-type gastric cancers, other histological types were also partially recognized and were recorded. The degree of lymphovascular invasion was scored as follows: none, minimal, moderate, and marked. Stromal features that were evaluated in the study included desmoplasia, myxoid change, and inflammation. The growth pattern at the advancing edge of the deeply invasive area was classified into two types: expanding and infiltrative types [23]. In addition, the growth pattern of the intramucosal area, which is usually different from that of the deeply invasive area, was evaluated separately; the pattern was identified as “permeative” when the neoplastic cells infiltrated between the normal pits or glands with no recognizable margin to the growth, and “expansile” when the growing margin was sharply delineated and the tumor had a well-defined margin at the advancing edge. In addition, tumor size in each case was measured separately in the mucosal and deeply invasive components in the following procedure. First, we took gross photographs of the mucosal surface and cut surfaces of the tumor, and then each section for histological examination was marked on a printed photograph. In advanced tumors, we took sections in 5-mm slices including the greatest dimension and the deepest penetration of tumor. Additional sections perpendicular to the section of the greatest dimension were taken to figure out the spread of the tumor, and were submitted for histological evaluation. In early cancers, entire tumor was cut in 3–4-mm slices parallel to the lesser curvature, and all the sections were submitted for histological evaluation. After histological evaluation, the cancerous area was marked on the gross photographs of the cut surface as well as the mucosal surface to demonstrate the spread of the tumor accurately. Finally, we measured the size of the mucosal and deeper invasive components separately, and the size ratios of the deeper invasive components to the intramucosal components were calculated. In tumors with central ulceration, the intramucosal component remained at the ulcer edge at least in a small amount, in which case the size of the intramucosal components was defined as the total size of the intramucosal tumor at the ulcer edge and ulceration.

### Immunohistochemical studies

Formalin-fixed paraffin-embedded tissue blocks were available for all 87 cases. To determine the tumor immunophenotype, immunohistochemical staining was performed using antibodies (clone, dilution, manufacturer) for mucin 2 (Muc2) (CLH2, 1:500, Novocastra Laboratories, Newcastle, UK), CD10 (56C6, 1:100, Novocastra Laboratories), mucin 5AC (Muc5AC) (CLH5, 1:500, Novocastra Laboratories), and mucin 6 (Muc6) (CCP58, 1:500, Novocastra Laboratories). Immunohistochemical staining was performed using a Ventana Benchmark XT autostainer (Ventana Medical Systems, Tucson, AZ) with the labeled streptavidin–biotin peroxidase method, and the signals were visualized with 3,3′-diaminobenzidine.

Cytoplasmic staining for mucin core proteins and apical membranous staining for CD10 were evaluated. The tumor was defined as positive for each marker when more than 10 % of the neoplastic cells were stained, as reported previously [24]. On the basis of the immunohistochemistry, tumors were categorized into the gastric (Muc5AC^+^ and/or Muc6^+^; Muc2^−^ and CD10^−^), intestinal (Muc2^+^ and/or CD10^+^; Muc5AC^−^ and Muc6^−^), mixed (Muc2^+^ and/or CD10^+^; Muc5AC^+^ and/or Muc6^+^), and null (all negative) types.

### Statistical analysis

The clinicopathology data were compared by Fisher’s exact test or the chi square test for categorical variables, and Student’s *t* test or the Mann–Whitney *U* test for continuous variables. Survival curves were calculated by the Kaplan–Meier method and were compared using the log-rank test. Multivariate Cox proportional hazard models were used to identify the variables that were associated with disease-specific and disease-free survivals. Differences were considered significant when the *P* value from the two-tailed test was less than 0.05. Statistical analyses were performed with Excel Statistics (SSRI, Tokyo, Japan).

## Results

### Clinical characteristics of *RHOA*-mutated diffuse-type gastric cancer

The clinical features of the *RHOA*-mutant and *RHOA* wild-type tumors are summarized in Table 1. The patients with *RHOA*-mutant tumors included 13 men and nine women, with a mean age of 65 years (range 40–84 years). The anatomic distribution of the tumors was as follows: upper third, 4 (18 %); middle third, 11 (50 %); and lower third, 7 (32 %). Tumor sizes ranged from 2.2 to 15 cm (mean 6.4 cm; median 6.0 cm). Endoscopically, all the early cancers had the appearance of a superficial depressed lesion. Most of the advanced cancers were Borrmann type 3 lesions (*n* = 13, 81 %), and the remaining ones were Borrmann type 4 lesions (*n* = 3, 19 %). Six tumors with mutant *RHOA* (27 %) were early cancers (T1), whereas the other 16 tumors (73 %) were advanced cancers (T2–T4). Nodal metastases were noted in 16 cases (73 %). These features were not significantly different from those of *RHOA* wild-type tumors (Table 1).

**Table 1:** Clinicopathological features of *RHOA*-mutant and *RHOA* wild-type diffuse-type gastric cancer

Characteristics	*RHOA* mutated (*n* = 22)	*RHOA* wild type (*n* = 65)	*P*
Sex			
Male	13 (59 %)	38 (58 %)	1
Female	9 (41 %)	27 (42 %)	
Mean age and range (years)	65 (40–84)	63 (30–85)	0.5462
Locus			
Proximal third	4 (18 %)	16 (25 %)	0.7894
Middle third	11 (50 %)	28 (43 %)	
Distal third	7 (32 %)	21 (32 %)	
Mean tumor size ± SD (mm)	6.4 ± 3.3	9.1 ± 8.1	0.1352
Macroscopic type			
Early cancer			
Superficially depressed type	6 (100 %)	12 (100 %)	1
Advanced cancer			
Borrmann type 2	0	2 (4 %)	0.5115
Borrmann type 3	13 (81 %)	36 (68 %)	
Borrmann type 4	3 (19 %)	15 (28 %)	
T stage			
T1a, T1b	6 (27 %)	12 (18 %)	0.4132
T2	2 (9 %)	2 (3 %)	
T3	3 (14 %)	16 (25 %)	
T4a, T4b	11 (50 %)	35 (54 %)	
N stage			
N0	7 (32 %)	27 (42 %)	0.4601
N1	3 (14 %)	6 (9 %)	
N2	4 (18 %)	10 (15 %)	
N3	8 (36 %)	22 (34 %)	
Peritoneal dissemination			
Present	5 (23 %)	15 (23 %)	1
Absent	17 (77 %)	50 (77 %)	
M stage (distant metastasis)			
Present	1 (5 %)	5 (8 %)	1
Absent	21 (95 %)	60 (92 %)	
Stage			
I	6 (27 %)	11 (17 %)	0.7457
II	5 (23 %)	19 (29 %)	
III	5 (23 %)	17 (26 %)	
IV	6 (27 %)	18 (28 %)	

*SD* standard deviation

### Histological features of *RHOA*-mutated diffuse-type gastric cancers

**Fig. 1:**
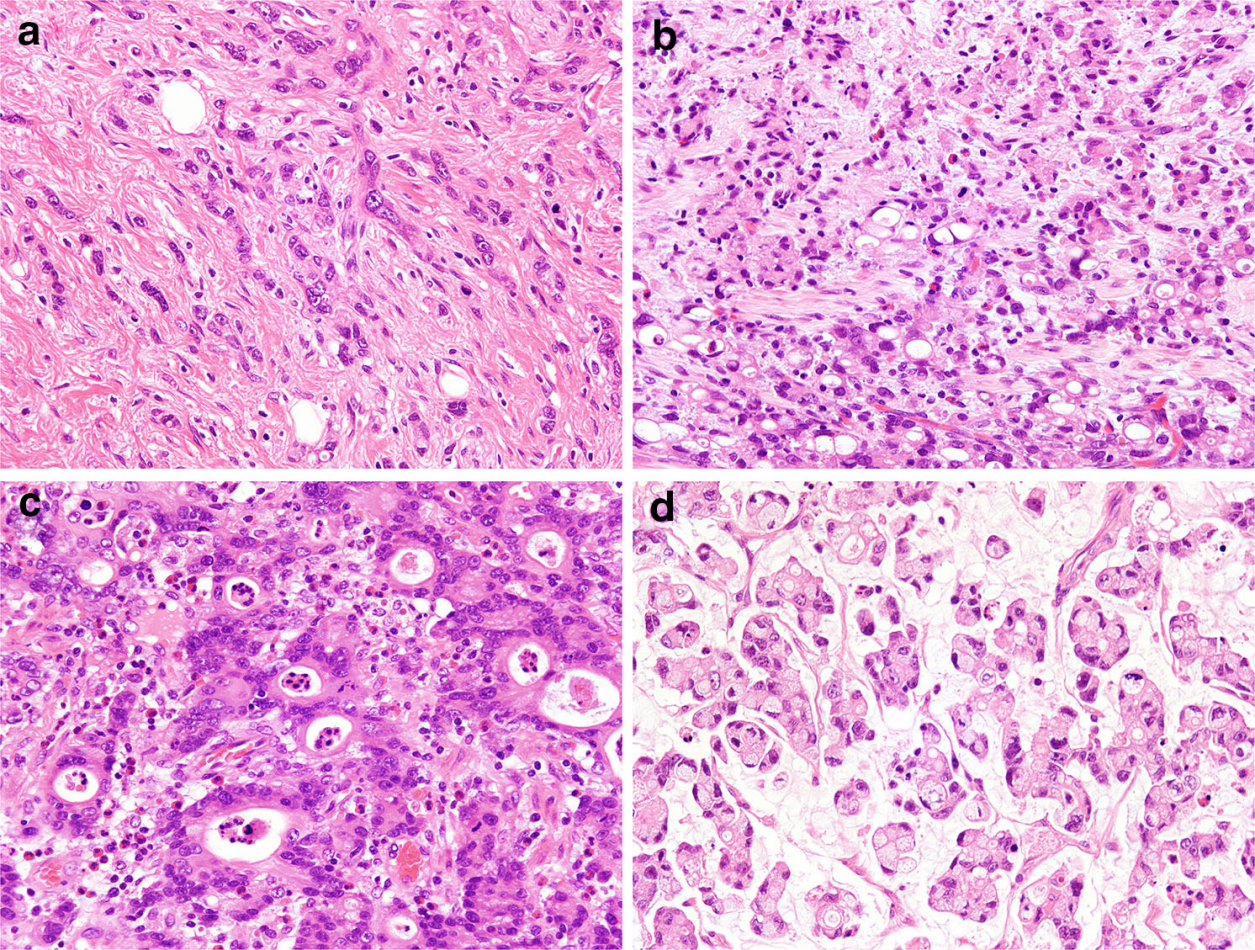
Histology of *RHOA*-mutated gastric cancers. Poorly cohesive carcinoma is the predominant component (**a**), including signet-ring cells in many cases (**b**). Focal tubular differentiation is frequently recognized (**c**), and a mucinous component may also be present (**d**)

The associations between the *RHOA* mutation and histological characteristics are summarized in Table 2. The histological type of the *RHOA*-mutated tumors was less frequently pure poorly cohesive carcinoma than for the *RHOA* wild-type tumors (27 % vs 46 %), although this did not reach statistical significance (*P* = 0.1201) (Fig. 1a). Signet ring cell carcinoma, a major variant of poorly cohesive carcinoma, was identified in 18 of the 22 *RHOA*-mutated tumors (82 %) and in 52 of the 65 *RHOA* wild-type tumors (80 %) (Fig. 1b). *RHOA*-mutated tumors showed focal tubular differentiation in 16 of the 22 cases (73 %), including three (14 %) with mucinous differentiation (Fig. 1c, d). *RHOA* wild-type tumors also had focal tubular and/or mucinous differentiation in approximately half of the cases. Tubular differentiation within the mucosal area was more frequently observed in *RHOA*-mutated tumors (16 of 22, 73 %) than in *RHOA* wild-type tumors (28 of 65, 43 %, *P* = 0.0254), whereas tubular differentiation in the submucosa or deeper area was noted at similar frequencies in the two groups (53 % vs 46 %, *P* = 0.7922). There were no significant differences in the stromal features and the extent of lymphovascular invasion and perineural invasion between the two groups. Notably, four *RHOA*-mutated tumors (18 %) and eight *RHOA* wild-type tumors (12 %, *P* = 0.4893) demonstrated carcinomatous lymphangiosis, which was characterized by prominent lymphatic involvement in the full thickness of the gastric wall with dilated lymphatics filled with neoplastic cells.

**Table 2:** Histological features of diffuse-type gastric cancers with or without *RHOA* mutation

Findings	*RHOA* mutated (*n* = 22)	*RHOA* wild type (*n* = 65)	*P*
Histological type			
Pure poorly cohesive carcinoma	6 (27 %)	30 (46 %)	0.1201
Poorly cohesive plus other types	16 (73 %)	35 (54 %)	
Presence of tubular component	16 (73 %)	33 (51 %)	0.0862
Intramucosal area	16 (73 %)	28 (43 %)	0.0254
Submucosal or deeper area	10 (53 %)	27 (46 %)	0.7922
Presence of mucinous component	3 (14 %)	9 (14 %)	1.0000
Stromal features			0.3575
Desmoplastic	18 (82 %)	58 (89 %)	
Inflammatory	9 (41 %)	13 (20 %)	
Myxoid	5 (23 %)	14 (22 %)	
Normal	4 (18 %)	6 (9 %)	
Lymphatic invasion			
Negative or minimal	12 (55 %)	39 (60 %)	0.8028
Moderate or marked	10 (45 %)	26 (40 %)	
Vascular invasion			
Negative or minimal	13 (59 %)	36 (55 %)	0.8082
Moderate or marked	9 (41 %)	29 (45 %)	
Carcinomatous lymphangiosis	4 (18 %)	8 (12 %)	0.4893
Perineural invasion	7 (32 %)	31 (48 %)	0.2226
Growth pattern			
Intramucosal area			
Permeative	13 (59 %)	19 (29 %)	0.0202
Expansile	9 (41 %)	46 (71 %)	
Submucosa or deeper area			
Infiltrative	19 (100 %)	58 (98 %)	1.0000
Expanding	0	1 (2 %)	
Ratio of deeply invasive to intramucosal size			
≥1.45^a^	6 (32 %)	34 (58 %)	0.0482
<1.45	13 (68 %)	25 (42 %)	

^a^Median of the ratios of deeply invasive to intramucosal size

**Fig. 2:**
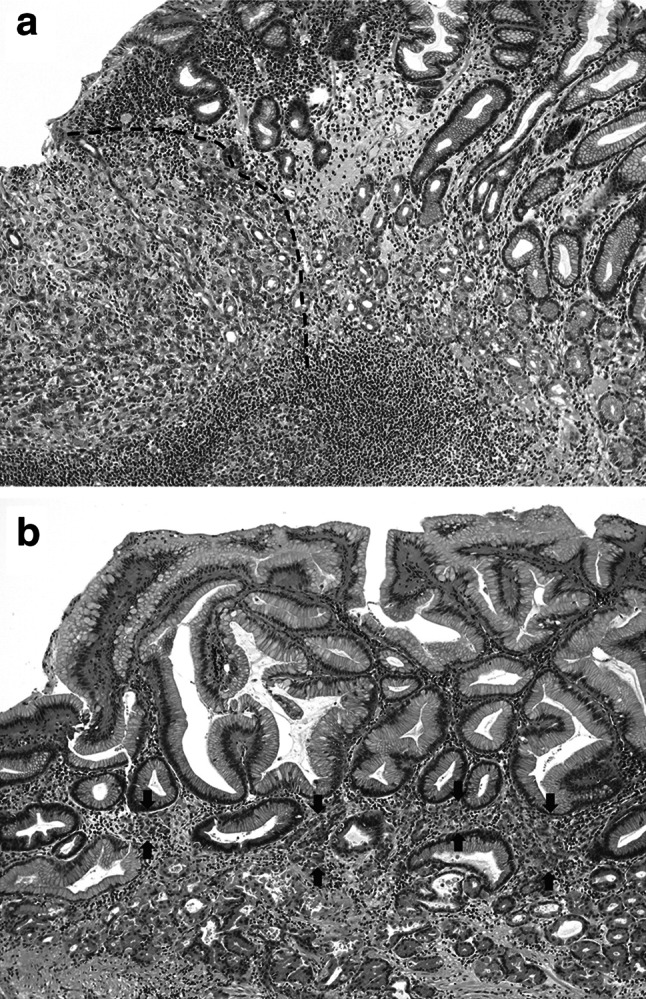
Growth patterns at the edge of the intramucosal component. The expansile pattern demonstrates destructive invasion with a relatively well-defined margin (indicated by the *dotted line*) at the advancing edge (**a**). In the permeative pattern, neoplastic cells infiltrate between the normal pits or glands in the middle layer of the lamina propria, with no recognizable margin to the growth (**b**). Neoplastic cells are indicated by *arrows*

**Fig. 3:**
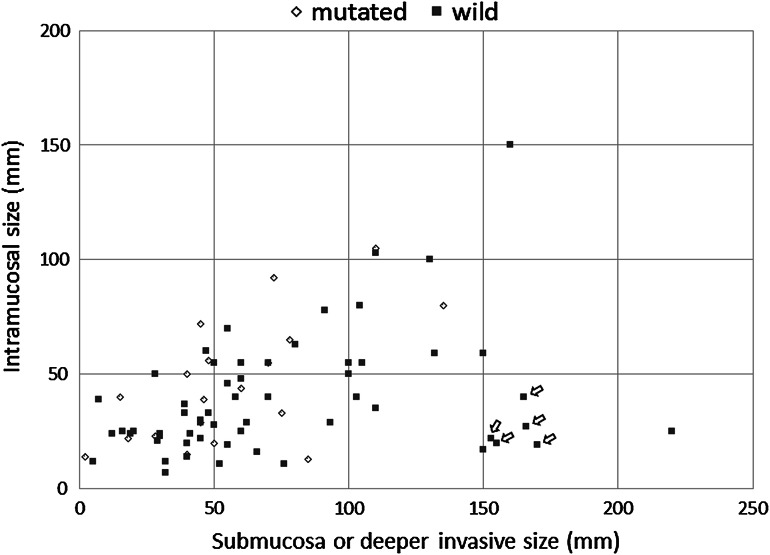
Correlation between the sizes of the intramucosal components and those of the submucosal or deeper areas of each case. *Arrows* at the lower right indicate cases of linitis plastica type cancer (*n* = 5)

*RHOA*-mutated tumors demonstrated distinct morphological features in terms of the growth pattern. An intramucosal permeative growth pattern was more frequently observed in *RHOA*-mutated tumors than in *RHOA* wild-type tumors, with a significant difference (59 % vs 29 %, *P* = 0.0202) (Fig. 2). The growth patterns in the deeply invasive area did not differ between the two groups, both of which usually demonstrated an infiltrative growth pattern. However, the size ratios of the submucosal or deeper invasive area to the intramucosal area were significantly lower in cases with *RHOA* mutation [less than 1.45 (median) in 68 %] than in those without *RHOA* mutation (less than 1.45 in 42 %, *P* = 0.0482). There was a significant difference in the ratios of deeply invasive to intramucosal size between the two groups when compared by the Mann–Whitney *U* test (*P* = 0.0308). Our cohort included five cases of linitis plastica type cancer, which is characterized as a leather bottle-like Borrmann type 4 tumor with relatively small intramucosal components in proportion to the extensive gastric wall involvement, and all of these cases were of the *RHOA* wild type (Fig. 3).

### Correlation of *RHOA* mutation with immunophenotype

On the basis of immunostains for gastric phenotypic markers (Muc5AC and Muc6) and intestinal phenotypic markers (Muc2 and CD10), the 22 *RHOA*-mutated tumors were classified as gastric type (*n* = 10, 45 %), mixed type (*n* = 8, 36 %), intestinal type (*n* = 1, 5 %), and null type (*n* = 3, 14 %). The 65 *RHOA* wild-type tumors were classified as gastric type (*n* = 31, 48 %), mixed type (*n* = 22, 34 %), intestinal type (*n* = 7, 11 %), and null type (*n* = 5, 7 %). There was no significant difference in the frequencies of each phenotype between the two groups.

### Patient outcome and prognostic factors

Follow-up information for 1–126.7 months (mean 46.8 months) was available for all of the cases. All the patients with stage I disease (*n* = 17) were alive without disease at the last contact (range 17–126.7 months; mean 78.1 months), except for one patient, who died of another disease. Patients with stage II–IV disease (*n* = 70) had either died of the disease (*n* = 33), were alive without the disease (*n* = 28), were alive with the disease (*n* = 6), or had died of another disease (*n* = 3). Noticeably, a single patient with *RHOA*-mutated early cancer (pT1b) developed pulmonary hypertension due to pulmonary tumor thrombotic microangiopathy that was caused by gastric cancer, which was revealed by video-assisted thoracic surgery and subsequent autopsy.

**Fig. 4:**
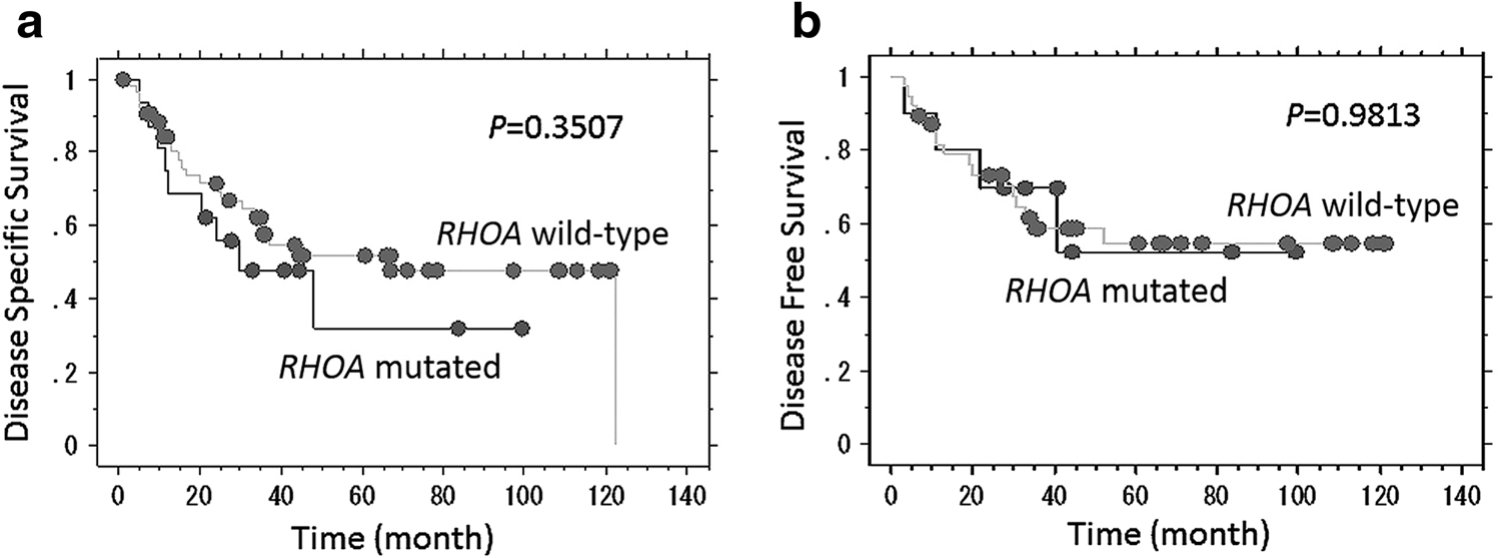
Kaplan–Meier survival plot according to *RHOA* mutation status among patients with the stage II–IV disease. *RHOA* mutation was not significantly associated with disease-specific survival (**a**) or disease-free survival (**b**) of patients with diffuse-type gastric cancer

A survival analysis was performed on the patients with stage II–IV disease. In univariate analyses, the Kaplan–Meier survival curves demonstrated that *RHOA* mutation was not significantly associated with disease-specific survival (*P* = 0.3507 by the log-rank test) or disease-free survival (*P* = 0.9813) (Fig. 4). Stage IV disease (vs stage II–III disease), the presence of lymphatic invasion, and lymph node metastasis were associated with decreased disease-specific survival (*P* < 0.0001, 0.0003, and 0.0004, respectively) and disease-free survival (*P* = 0.0029, 0.0016, and 0.0111, respectively). Other features, including gender, tumor size, T stage, venous invasion, and perineural invasion, were not associated with differences in disease-specific survival or disease-free survival. In multivariate analyses, *RHOA* mutation was not a significant prognostic factor.

## Discussion

Comprehensive genomic analyses have recently increased our understanding of gastric cancer [9–11]. The largest study to date, conducted by The Cancer Genome Atlas (TCGA) project, proposed a molecular classification that divided gastric cancer into four subtypes: tumors positive for Epstein–Barr virus, microsatellite-unstable tumors, genomically stable tumors, and tumors with chromosomal instability [10]. Genomically stable tumors, which nearly correspond to diffuse-type cancers in terms of histological features, were characterized by mutations of *RHOA* or *CLDN18*–*ARHGAP6*/*ARHGAP26* fusions in addition to the well-known mutations of *CDH1*. Within the genomically stable subgroup, 30 % of the cases had either *RHOA* or *CLDN18*–*ARHGAP* alterations. Furthermore, analyses of the gene expression status in the RhoA-signaling pathways suggested that these genomic alterations resulted in the activation of the RhoA-driven pathways [10]. These results obtaiend by the TCGA project are consistent with our previous study demonstrating the recurrent *RHOA* mutations exclusively in diffuse-type gastric cancer (25 % of the cases) [9]. Our small interfering RNA knockdown and rescue experiments showed growth-promoting effects of mutant *RHOA*, suggesting a gain-of–function role for *RHOA* mutations in progression of diffuse-type gastric cancers [9]. On the other hand, another report with a functional study suggested that mutant *RHOA* might cause defective RhoA signaling, which would promote escape from anoikis, an important early step in the carcinogenesis of diffuse-type gastric cancers [11]. The details of the functional consequences of *RHOA* mutation in diffuse-type gastric cancer still remain largely unknown, and further research will be required to provide a more thorough understanding of the role of *RHOA* mutation in diffuse-type gastric cancer.

This study was the first to perform a thorough clinicopathology review of *RHOA*-mutated gastric cancers. From our observations, advanced *RHOA*-mutated tumors were characterized as Borrmann type 3 lesions (81 %) that developed in the middle third (50 %) or distal third (32 %) of the stomach. Histologically, tubular differentiation was frequently observed (73 %) in addition to predominant poorly cohesive carcinoma. Notably, *RHOA*-mutated tumors more frequently showed permeative growth patterns at the edge of the mucosal area than did *RHOA* wild-type tumors, with a significant difference. In addition, the size ratio of the mucosal components to the deeply invasive components was significantly higher in tumors with *RHOA* mutation than in those without *RHOA* mutation. Lnitis plastica type cancers, which are typically characterized as a leather bottle-like (Borrmann type 4) appearance, relatively small mucosal lesions in proportion to the extensive spread in the gastric wall, and histologically pure poorly cohesive carcinoma, were of the *RHOA* wild type in our cohort.

*RHOA* mutation may contribute to the intramucosal permeative growth pattern, potentially resulting in a relatively large mucosal component in proportion to the deeply invasive area. First, RhoA is a critical regulator of actin–myosin-dependent cell contractility and cellular motility [12, 13, 21]. In particular, RhoA signaling drives amoeboid motility, which is characterized by protease-independent cellular movement, i.e., via propulsive squeezing through gaps of the extracellular matrix using an actomyosin-related contractile force [25, 26]. Second, *RHOA* mutation has been reported to be an early event in carcinogenesis, suggesting that tumor cells harbor the mutation at the early stage in the mucosa [9]. Therefore, it is possible that the intramucosal permeative growth pattern might reflect alterations in RhoA signaling. In addition, RhoA alterations could be associated with lymphovascular invasion, causing carcinomatous lymphangiosis or pulmonary tumor thrombotic microangiopathy in some extreme cases because RhoA is important in the transendothelial migration of neoplastic cells [27–29]. This hypothesis, however, remains speculative because there is a lack of data supporting an association between *RHOA* mutation and lymphovascular invasion in this study. Further functional studies are necessary to clarify the role of *RHOA* mutation in diffuse-type gastric cancers, particularly to develop therapeutic agents that target mutant RhoA.

*RHOA* mutation did not appear to have a significant impact on the survival in this study. The relatively small number of cases was an inherent limitation of our study. A larger sample size is necessary to verify the prognostic importance of the *RHOA* mutation. In addition, it is also important to include analyses of other genomic alterations that affect the RhoA-signaling pathway, such as *CLDN18*–*ARHGAP* fusion, which has been predicted to alter RhoA-driven pathways as well as *RHOA* mutation [10]. Furthermore, TCGA data have suggested that there may be additional events within the genome-stable subgroup that result in RhoA-signaling activation because alterations in the RhoA pathway are present in cases without *RHOA* mutation or *ARHGAP* fusion as well [10]. Therefore, although *RHOA* mutation may not be a significant prognostic factor in isolation, analyses that include other genetic alterations involving RhoA pathways would better clarify the significance of the RhoA-signaling alterations.

In summary, advanced diffuse-type gastric cancers with *RHOA* mutation were characterized as Borrmann type 3 tumors with relatively large intramucosal components in proportion to deeply invasive components, frequent tubular differentiation in addition to predominant poorly cohesive carcinoma, and an intramucosal permeative growth pattern. Although *RHOA* mutation did not significantly impact the survival in the relatively small number of patients, further studies that include analyses of other alterations involving RhoA-signaling pathways, as well as a larger sample size of cases, are necessary to determine the significance of alterations in the RhoA-signaling pathway.
